# Response Inhibition and Interference Control in Obsessive–Compulsive Spectrum Disorders

**DOI:** 10.3389/fnhum.2014.00419

**Published:** 2014-06-11

**Authors:** Laura S. van Velzen, Chris Vriend, Stella J. de Wit, Odile A. van den Heuvel

**Affiliations:** ^1^GGZ InGeest, Amsterdam, Netherlands; ^2^Neuroscience Campus Amsterdam (NCA), Amsterdam, Netherlands; ^3^Department of Psychiatry, VU University Medical Center, Amsterdam, Netherlands; ^4^Department of Anatomy and Neurosciences, VU University Medical Center, Amsterdam, Netherlands

**Keywords:** response inhibition, obsessive–compulsive disorder, Tourette’s syndrome, trichotillomania, attention-deficit hyperactivity disorder, interference control

## Abstract

Over the past 20 years, motor response inhibition and interference control have received considerable scientific effort and attention, due to their important role in behavior and the development of neuropsychiatric disorders. Results of neuroimaging studies indicate that motor response inhibition and interference control are dependent on cortical–striatal–thalamic–cortical (CSTC) circuits. Structural and functional abnormalities within the CSTC circuits have been reported for many neuropsychiatric disorders, including obsessive–compulsive disorder (OCD) and related disorders, such as attention-deficit hyperactivity disorder, Tourette’s syndrome, and trichotillomania. These disorders also share impairments in motor response inhibition and interference control, which may underlie some of their behavioral and cognitive symptoms. Results of task-related neuroimaging studies on inhibitory functions in these disorders show that impaired task performance is related to altered recruitment of the CSTC circuits. Previous research has shown that inhibitory performance is dependent upon dopamine, noradrenaline, and serotonin signaling, neurotransmitters that have been implicated in the pathophysiology of these disorders. In this narrative review, we discuss the common and disorder-specific pathophysiological mechanisms of inhibition-related dysfunction in OCD and related disorders.

## Introduction

Response inhibition, the ability to suppress pre-potent behavior that is inappropriate or no longer required, is critical for goal-directed behavior in everyday life (Chambers et al., 2009). Over the past decades, researchers have shown increased interest in response inhibition. Response inhibition is considered an operationalization of certain aspects of impulsivity and compulsivity (Bari and Robbins, 2013). Impulsivity is commonly defined as a tendency to act on impulses, acts performed immediately and without voluntary control, whereas compulsivity is the tendency to repeat specific behavior and to be unable to inhibit the behavior even when it is no longer appropriate (Bari and Robbins, 2013). Due to the importance of response inhibition in everyday life, many neuropsychological paradigms have been developed to probe inhibitory performance. In these paradigms, subjects are asked to respond to a target stimulus, but withhold this response to irrelevant or distracting stimuli, or distracting stimulus characteristics (Nigg, 2000).

**Figure 1:**
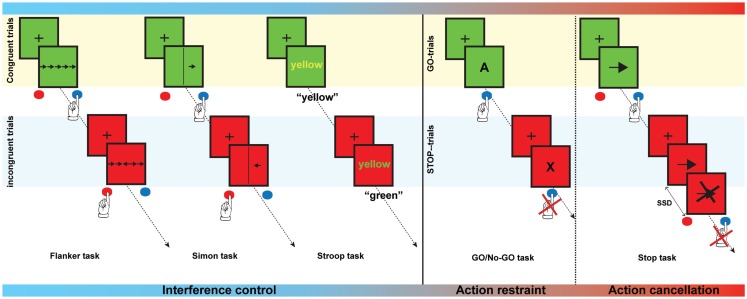
**Examples of interference control and motor response inhibition tasks**. The *Flanker task* is a test in which subjects are asked to respond to a target stimulus by pressing a button to indicate the direction of the target stimulus. The target, however, is flanked by non-target distracter stimuli, which are presented in the same or in the opposite direction as the target (congruent and incongruent trials, respectively). During a *Simon task*, participants are asked to press a button depending on the orientation of the arrow, irrespective of the location of the arrow. Orientation and location can either be congruent of incongruent. In the *Stroop task* names of colors are presented in either the same (congruent) or a different color (incongruent). Subjects are instructed to name to color of the word but not the word itself. In the *Go/No-go task*, subjects need to respond as fast as possible when letters are presented (Go-trials), but must withhold the response when a certain letter (e.g., “X”) is presented (Stop-trials). In a *Stop-signal task*, the participant is asked to respond as fast as possible by pressing a button to a stimulus (Go-trials) that is presented. On a minority of trials, a stop-signal is presented and the subject is asked to suppress the response when the stop-signal occurs. Task demands gradually increase from interference control to action cancelation.

Response inhibition is not a unitary construct and consists of motor response inhibition and interference control. Motor response inhibition involves the inhibition of pre-potent and automatic motor responses, and can be further differentiated into action restraint (or action suppression) and action cancelation (Schachar et al., 2007). The Go/No Go task (Donders, 1969) is considered to probe action restraint, whereas the Stop-signal task (Logan, 1994) measures action cancelation (see Figure 1 for a description of these tasks). Interference control on the other hand, refers to the cognitive control needed to prevent interference due to competition of relevant and irrelevant stimuli or stimulus characteristics (Nigg, 2000). Several tasks including the Stroop task, the Flanker task and the Simon task are measures of interference control (see Figure 1). It has been proposed that the inhibitory load is highest in the Stop-signal task, as the response that needs to be suppressed has already been initiated (Schachar et al., 2007). Contrary to the motor response inhibition tasks, interference control tasks may also rely on response selection processes (Nee et al., 2007). It has been suggested that interference control, action restraint, and action cancelation represent early, intermediate, and late processes of response inhibition, respectively (Sebastian et al., 2013a).

The symptoms of obsessive–compulsive and related disorders within the impulsive–compulsive spectrum are characterized by a failure to inhibit certain behaviors, e.g., washing hands, pulling hair, motor tics, or impulsive actions. Response inhibition might therefore be a suitable measure to investigate the neural substrates of these shared symptoms. In this narrative review, we will provide an overview of studies that have examined the neuroanatomical and functional underpinnings of response inhibition impairment in healthy subjects and patients with these disorders but this review by no means constitutes an exhaustive account of the current literature. We will focus on the shared mechanisms that may underlie the inhibitory dysfunction and symptoms of these disorders. Pharmacological and genetic alterations are also addressed and focus on the dopamine, serotonin, and noradrenalin system. We acknowledge that other neurotransmitters, such as glutamate and gamma-Aminobutyric acid, are also important for response inhibition and the pathophysiology obsessive–compulsive and related disorders (MacMaster et al., 2003; Turner et al., 2003; van Minnen et al., 2003; DeVito et al., 2005; Starck et al., 2008; Silveri et al., 2013), but discussion of all these neurotransmitters would considerably lengthen this review.

## Neural Correlates of Response Inhibition in Healthy Controls

### Neuroimaging of response inhibition

Neuroimaging studies in healthy controls have revealed the neural substrates of response inhibition [for excellent reviews, see Robbins (2007), Chambers et al. (2009), and Aron (2011)]. While major contributions to our understanding of response inhibition come from electrophysiological studies, in this review, we will focus on neuroimaging studies. Readers interested in the electrophysiology of response inhibition are directed to Huster et al. (2013).

In brief, response inhibition activates a network of mainly right lateralized frontal brain areas. The inferior frontal gyrus (IFG) and pre-supplementary motor area (pre-SMA) are key components (Aron et al., 2003b; Chambers et al., 2006; Floden and Stuss, 2006; Cai et al., 2012), and the neural stop-signal is then sent from these frontal areas to the motor cortex through cortico-striatal–thalamic–cortical (CSTC) projections (Chambers et al., 2009).

The subcomponents of response inhibition are found to depend on overlapping, yet distinct, brain areas. Interference inhibition, action restraint and action cancelation are all associated with activation of the IFG, anterior insula, anterior cingulate cortex (ACC), dorsolateral prefrontal cortex (DLPFC), pre-SMA, and parietal regions (Wager et al., 2005; Nee et al., 2007; Sebastian et al., 2013b). When inhibitory task load increases, activation of frontal–striatal regions increases and additional inhibition-related brain areas are recruited (Blasi et al., 2006; Swick et al., 2011; Sebastian et al., 2013b). However, each task recruits distinct brain areas as well, depending on the unique cognitive processes that they represent. Regions involved in response selection, including the parietal cortex, for instance, are activated to a greater extent during interference control tasks and action restraint (Rubia et al., 2001; Sebastian et al., 2013b).

### Neurotransmitters in response inhibition

In addition to differences in neural activation, differences in the neurotransmitter systems underlying interference control, action restraint, and action cancelation have also been observed. Current studies suggest that interference control is dependent on serotonin and dopamine neurotransmission. Depletion of serotonin and dopamine has been shown to decrease interference effects on incongruent trials, and thus improve performance, during the Stroop task (Schmitt et al., 2000; Scholes et al., 2007). Decreases in serotonin may improve performance by increasing arousal and attention (Scholes et al., 2007). Increased activation of the DLPFC and ACC was observed during performance of a Stroop task after serotonin depletion (Horacek et al., 2005). Stroop performance was positively correlated with serotonin transporter (SERT) binding in the right DLPFC as well (Madsen et al., 2011). However, contradicting results have also been reported, as neuroimaging studies showed that decreased dopamine transporter (DaT) binding in the striatum in women (Mozley et al., 2001) and decreased postsynaptic striatal D_2_-receptor availability was associated with poor performance on a Stroop task (Volkow et al., 1998). Administration of a dopamine D_2_ agonist decreased interference, and thereby improved performance on the Stroop task (Roesch-Ely et al., 2005). Based on the available literature in healthy subjects it seems that both serotonin and dopamine are important for interference control.

Action restraint (Go/No Go task) seems to be primarily mediated by serotonin [for a review of evidence, see Eagle et al. (2008)]. Serotonin depletion has been shown to decrease activation of the IFG during inhibition and decreases activation of the medial prefrontal lobe during error monitoring in the Go/No Go task (Rubia et al., 2005a; Evers et al., 2006). Administration of a serotonin 2C receptor agonist (Anderson et al., 2002) or mirtazapine (Vollm et al., 2006), which acts on both the noradrenalin and serotonin system, increased inhibition-related activation of the right IFG. Nevertheless, dopamine may play a role in action restraint as well since methylphenidate, a dopamine re-uptake inhibitor, improved performance on the Go/No Go task and led to decreased task-related striatal activation (Vaidya et al., 1998).

Several lines of evidence support an important role for dopamine in action cancelation. Increased Dopamine D_2/3_-receptor availability in the striatum is associated with better Stop-signal task performance, i.e., shorter stop-signal reaction time (SSRT), and correlates positively with inhibition-related activation of the dorsal caudate and putamen (Ghahremani et al., 2012). Also, administration of a D_2_-receptor agonist improved action cancelation (Nandam et al., 2013). Administration of methylphenidate and atomoxetine, which both target the dopamine and noradrenalin system, by inhibiting the re-uptake from the synaptic cleft, decreased SSRT in humans and in animals, raising the possibility that noradrenalin is involved in motor response inhibition as well (Chamberlain et al., 2006b; Eagle et al., 2007; Bari et al., 2009; Nandam et al., 2011). Serotonin does not seem to mediate performance of the Stop-signal task, as use of selective serotonin re-uptake inhibitors (SSRIs) and serotonin depletion did not affect action cancelation in humans or in animals (Clark et al., 2005; Chamberlain et al., 2006b; Bari et al., 2009; Eagle et al., 2009; Drueke et al., 2010).

To summarize, interference control seems to be mediated by both serotonin and dopamine. Action restraint seems to be predominantly mediated by serotonin, whereas action cancelation seems to be mediated by dopamine and noradrenalin. More detailed information on the neuropharmacology of response inhibition is provided by Dalley and Roiser (2012) and Bari and Robbins (2013).

### Genes in response inhibition

Several gene-association studies have examined the relationship between genes involved in the dopaminergic and serotonergic systems and response inhibition. Genetic polymorphisms in the dopamine D_4_-receptor gene (DRD4), associated with reduced functional activity (Asghari et al., 1995), have been related to decreased performance on the Stop-signal task (Congdon et al., 2008), although conflicting results have also been reported (Kramer et al., 2009).

The DRD2 gene codes for the dopamine receptor D_2_. The presence of a TaqIA allele, which has been linked to decreased availability of striatal D_2_-receptors (Thompson et al., 1997), was associated with poor response inhibition in the Stop-signal task (White et al., 2008). A second polymorphism, which has been associated with decreased cortical and thalamic D_2_-receptor availability (Hirvonen et al., 2009), was also related to poor action cancelation (Colzato et al., 2010).

Genetic polymorphisms that increased expression of the DaT have been associated with impaired performance on an interference control task (Cornish et al., 2005) and decreased brain activation in the STN and pre-SMA during action cancelation (Congdon et al., 2009). Furthermore, two novel single nucleotide polymorphisms in the DaT gene predicted individual SSRT (Cummins et al., 2012) and genotype of one of these polymorphisms predicted activation of frontal areas and the caudate nucleus during task performance.

Polymorphisms in catechol-*O*-methyltransferase (COMT) and monoamine oxidase A (MAO-A) genes, coding for enzymes playing a role in neurotransmitter metabolism, have been associated with normal variations in inhibition-related activity as well. COMT is involved in the degradation of dopamine and noradrenalin and MAO-A is involved in degradation of dopamine, noradrenalin, and serotonin. A polymorphism of COMT with decreased function (Chen et al., 2004), was associated with increased activation of the IFG during action cancelation (Congdon et al., 2009) and decreased interference inhibition (Solis-Ortiz et al., 2010), although conflicting results have also been reported (Kramer et al., 2007). Polymorphisms of the MAO-A gene, which increase MAO-A activity, were associated with increased activity in the right IFG and ACC and decreased activity in the superior parietal cortex during action restraint (Passamonti et al., 2006).

Gene-association studies have focused on genes involved in serotonergic transmission as well. Serotonin synthesis in the brain is regulated by tryptophan-hydroxylase-2 (TPH-2) (Walther and Bader, 2003). Individuals homozygous for the T-allele of a polymorphism in the TPH-2 gene showed increased SSRT in the Stop-signal task (Stoltenberg et al., 2006). A second study found that two other polymorphisms in the TPH-2 gene were associated with reduced brain activity during action restraint in an EEG study (Baehne et al., 2009). Lastly, Osinsky et al. (2009) found that a polymorphism located in the promotor region of the TPH-2 gene, affected reaction time during performance of a Stroop task. Interpretation of these findings is, however, challenging as it is uncertain how these polymorphisms affect serotonin levels.

Polymorphisms in the SERT gene (SLC6A4) that decrease the rate of re-uptake from the synaptic cleft, were associated with decreased interference inhibition (Holmes et al., 2010), but not to action cancelation (Clark et al., 2005). Participants with a decreased function polymorphism in SERT also showed increased rostral ACC activation in response to errors and decreased activation of the dorsal ACC in response to conflict during the Flanker task (Holmes et al., 2010).

### Intermediate summary

In summary, current evidence suggests that response inhibition is dependent on brain areas in the CSTC circuits and activation in these circuits increases with increasing inhibitory load. Proper function of these CSTC circuits depends on a complex interplay between dopamine, serotonin, and noradrenalin, although the weight of their importance may differ between the subcomponents of response inhibition. While reducing levels of serotonin and dopamine appears to ameliorate interference control, increasing dopamine levels appears to ameliorate action restraint and action cancelation. Gene-association studies have primarily reported that polymorphisms associated with decreased dopamine signaling are also associated with decreased motor response inhibition performance.

Structural and functional alterations in CSTC circuits and altered serotonin, noradrenalin, and dopamine transmission may underlie response inhibition deficits in obsessive–compulsive disorder (OCD) patients and in patients with related disorders.

## Obsessive–Compulsive Disorder and Inhibition

Obsessive–compulsive disorder is an anxiety disorder that affects 2–3% of the population and causes severe impairment in social and occupational functioning (Ruscio et al., 2010). The disorder is characterized by distress- and anxiety provoking obsessions (repetitive intrusive thoughts) and compulsions (repetitive ritualistic behavior), which are performed to diminish anxiety (American Psychiatric Association, 2013). These symptoms are common, as more than 25% of the population experiences sub-clinical obsessions or compulsions in their lives (Ruscio et al., 2010). Pharmacotherapy for OCD consists mainly of SSRIs, which suggests involvement of the serotonin system in the pathophysiology of the disorder. Nevertheless, an estimated 40–60% of patients does not respond to this treatment and require additional treatment with atypical antipsychotics, which affects both the serotonergic and dopaminergic system (Denys et al., 2004a; Fineberg et al., 2005). Neuroimaging studies have strengthened the notion of serotonergic dysfunction in OCD by providing evidence for reduced availability of SERTs in the midbrain, thalamus, and brainstem and reduced availability of serotonin 2A receptors in prefrontal, parietal, and temporal brain regions (Hesse et al., 2005; Perani et al., 2008). Abnormalities in the dopamine system have also been observed in OCD patients, such as increased DaT levels in the striatum and reduced availability of the D_1_- and D_2_-receptors in the striatum (Kim et al., 2003; Denys et al., 2004b; van der Wee et al., 2004; Olver et al., 2009).

In the past several years, research interest has focused on response inhibition as a model of OCD symptoms (Chamberlain et al., 2005). In support of this, deficits in interference control, e.g., increased reaction times during incongruent trials, have been described in OCD (Bannon et al., 2002; Penades et al., 2007; Nabeyama et al., 2008; Nakao et al., 2009; Schlosser et al., 2010). A number of studies have used interference control paradigms in OCD research during functional neuroimaging (see Table 1; Fitzgerald et al., 2005; Nakao et al., 2005a, 2009; van den Heuvel et al., 2005; Viard et al., 2005; Nabeyama et al., 2008; Woolley et al., 2008; Page et al., 2009; Schlosser et al., 2010; Huyser et al., 2011; Rubia et al., 2011a; Marsh et al., 2013). Some studies reported hyperactivation of the ACC in adults and children with OCD following errors and interference control (Fitzgerald et al., 2005; Huyser et al., 2011), while others reported hypoactivation of the ACC (Nakao et al., 2005a; Rubia et al., 2011a). Altered inhibition-related brain activation has also been observed in the pre-SMA (Fitzgerald et al., 2005; Rubia et al., 2011a) and insular cortex (Huyser et al., 2011). Increased activation in frontal–striatal regions, including the IFG and putamen, was seen in OCD patients during performance of a Simon task (Marsh et al., 2013).

**Table 1:** **Overview of fMRI studies that have used interference control tasks in obsessive–compulsive disorder**.

Study	Task	Age group	Participants	Medication and co-morbidities	Contrast	Findings in OCD patients
Fitzgerald et al. (2005)	Flanker task	Adults	8 OCD patients (2 f) 7 Healthy controls (2 f)	Three patients were treated with SSRI’s, one with benzodiazepines and one received antipsychotic medication Three patients met criteria for depression, two for dysthymia. No severe medical conditions, neurological disorder, or head injury	E > Corr IC > C	↑ Rostral ACC (+correlation with severity of symptoms) ↓ R. pre-SMA ↑ Bilateral caudate nucleus
Huyser et al. (2011)	Flanker task	Children/adolescents	25 OCD patients (16 f) 25 Healthy controls (16 f)	Medication-free for at least 2 weeks prior to participation Forty-eight percent of patients had co-morbid anxiety disorder, 12% co-morbid affective disorders, 12% ADHD/ODD, and 8% tic disorders	E > Corr IC > C	↑ ACC, insula ↑ Bilateral insula
Nakao et al. (2005a)	Stroop task	Adults	24 OCD patients (15 f) 14 Healthy controls (9 f)	Medication-free for at least 2 weeks prior to participation No co-morbid axis-I disorders, no severe medical condition, neurological disorder, head injury, or substance abuse	IC > C	↑ R. frontal lobe ↓ Bilateral ACC, temporal lobe, R. caudate nucleus
Nabeyama et al. (2008)	Stroop task	Adults	11 OCD patients (7 f) 19 Healthy controls (11 f)	Medication-free for at least 2 weeks prior to participation Co-morbid disorders unreported	IC > C	↓R. ACC, R. cerebellum
Woolley et al. (2008)	Motor Stroop task	Children/adolescents	10 OCD patients (0 f) 9 Healthy controls (0 f)	Eight patients treated with an SSRI, five treated with CBT No co-morbid axis-I disorder, neurological disorder, head injury, and severe medical condition	IC > C	↓ R. middle temporal gyrus, bilateral cerebellum
Page et al. (2009)	Motor Stroop task	Adults	10 OCD patients (0 f) 11 Healthy controls (0 f)	Medication-free Two patients met criteria for dysthymic disorder, three previously met criteria for depression, and one previously met criteria for alcohol dependence	IC > C	↑ L. Cerebellum, L. posterior cingulate ↓ Bilateral precuneus, R. temporal gyrus L. temporo-parietal junction
Nakao et al. (2009)	Stroop task	Adults	17 OCD patients: duration of illness <10 years (12 f) 15 OCD patients: duration of illness >10 years (8 f) 16 Healthy controls (7 f)	Medication-free for at least 2 weeks prior to participation No co-morbid axis-I disorder, no severe medical condition, neurological disorder, head injury, or substance abuse	IC > C	↓ R. caudate, cerebellum in patients with disease duration <10 years compared with patients with longer disease duration and controls
Schlosser et al. (2010)	Stroop task	Adults	21 OCD patients (16 f) 21 Healthy controls (16 f)	Medication-free for at least 2 days prior to participation No co-morbid axis-I disorder, no psychosis, or neurological disorder	IC > C IC	↑ Bilateral DLPFC ↑ Bilateral superior frontal gyri, dorsal ACC, left precentral gyrus, right superior parietal lobe, and right inferior parietal
van den Heuvel et al. (2005)	Stroop task	Adults	18 OCD patients (12 f) 19 Controls (9 f)	Medication-free for at least 4 weeks prior to participation No neurological illness, other psychiatric disorders	IC > C	↑ R. precuneus, L. parahippocampal gyrus L. rostral brainstem
Viard et al. (2005)	Conflict task	Adults	12 OCD patients (5 f) 15 Healthy controls (4 f)	Eleven patients were treated with SSRI’s, one also with a TCA No co-morbid disorders, no severe medical condition, neurological disorder, or head injury	IC > C	No difference in brain activation
Marsh et al. (2013)	Simon task	Adults	22 OCD patients (11 f) 22 Healthy controls (11 f)	Medication-free Five patients had a lifetime history of depression	IC > C	↑ R. IFG, Insula, and putamen
Rubia et al. (2011a)	Simon task	Children/adolescents	10 OCD patients (0 f) 20 Healthy controls (0 f)	Eight patients were treated with SSRI’s; five patients with CBT No co-morbid psychiatric disorders, no history of learning disabilities, or substance abuse	IC > oddball	↓ R. pre-SMA, ACC, superior parietal cortex

*ADHD, attention-deficit hyperactivity disorder; C, congruent trials; CBT, cognitive behavioral therapy; corr, correct trials; DLPFC, dorsolateral prefrontal cortex; E, error trials; f, female; IC, incongruent trials; ODD, oppositional defiant disorder; OFC, orbitofrontal cortex; SMA, supplementary motor area; SSRI, selective serotonin re-uptake inhibitor; STG, superior temporal gyrus; TCA, tricyclic anti-depressant*.

Abnormalities in activation during interference control tasks have also been observed at a network level. Schlosser et al. (2010) used dynamic causal modeling (DCM) to examine functional connectivity in a fronto-cingulate network during performance on a Stroop task, and found increased connectivity between the DLPFC and ACC in OCD patients compared with healthy controls. Increased functional connectivity between the putamen and the inferior parietal cortex, caudate, thalamus, and frontal areas was observed in patients during performance of a Simon task (Marsh et al., 2013).

Impaired action cancelation and action restraint has been described for OCD (Chamberlain et al., 2007b; Penades et al., 2007); patients showed increased SSRT (i.e., slower inhibition) in the Stop-signal task and higher error rates on the Go/No Go task compared with healthy control subjects. Deficits in motor response inhibition were also observed in unaffected first-degree relatives of OCD patients (Chamberlain et al., 2007b; Menzies et al., 2007), suggesting that motor response inhibition may be considered an endophenotype [a trait that is heritable and co-segregates with the illness in families (Gottesman and Gould, 2003)] of OCD patients.

Structural neural correlates of impaired motor response inhibition in OCD patients have been identified. Deficits in action cancelation in OCD patients and first-degree relatives were associated with increased gray matter volume in the ACC, putamen, caudate, amygdala, parietal areas, and the cerebellum, and decreased gray matter volume in the OFC, IFG, ACC, premotor cortex, and regions in the temporal cortex (Menzies et al., 2007).

Functional neural correlates of motor response inhibition impairments in OCD have also been identified (see Table 2; Maltby et al., 2005; Roth et al., 2007; Woolley et al., 2008; Page et al., 2009; Rubia et al., 2010; de Wit et al., 2012; Kang et al., 2012). Decreased task-related activation is seen in the CSTC circuits during inhibition in OCD patients (Roth et al., 2007; Woolley et al., 2008; Page et al., 2009; Rubia et al., 2010; de Wit et al., 2012; Kang et al., 2012), although, one study reported increased activation of these regions (Maltby et al., 2005). In the largest study to date, de Wit et al. (2012) found decreased activation of the IFG and inferior parietal cortex during inhibition in unmedicated OCD patients and increased activation of the left pre-SMA. This pre-SMA hyperactivation was present in their unaffected siblings as well. Activation of the pre-SMA correlated negatively with SSRT in patients and siblings, indicating that hyperactivation of the pre-SMA may be considered a compensatory mechanism. Overall, the most consistent finding is decreased activation of the DLPFC, IFG, striatum, and thalamus in OCD patients during inhibition (Roth et al., 2007; Woolley et al., 2008; Page et al., 2009; Rubia et al., 2010; de Wit et al., 2012).

**Table 2:** **Overview of fMRI studies that have used response inhibition paradigms in obsessive–compulsive disorder**.

Study	Task	Age group	Participants	Medication and co-morbidities	Contrast	Findings in OCD patients
Maltby et al. (2005)	Go/No-go task	Adults	11 OCD patients (7 f) 11 Healthy controls (7 f)	Medication free; OCD is primary diagnosis, six patients met criteria for one other axis-I disorder No psychosis, neurological disorder, head injury, and substance abuse	FS > Go SS > Go	↑ Lateral prefrontal cortex, ACC, lateral OFC, caudate, thalamus during failed, and successful inhibition
Roth et al. (2007)	Go/No-go task	Adults	12 OCD patients (7 f) 14 Healthy controls (8 f)	Six patients treated with an SSRI Two patients met criteria for depression, one for social phobia. No neurological disorder, head injury, severe medical condition, or substance abuse	No Go > Go	↓ R. IFG, R. middle frontal gyrus
Woolley et al. (2008)	Stop-signal task	Children/adolescents	10 OCD patients (0 f) 9 Healthy controls (0 f)	Eight patients treated with an SSRI, five treated with CBT No comorbid axis-I disorder, neurological disorder, head injury, and severe medical condition	SS > FS FS > Go	↓ R. OFC, thalamus, basal ganglia ↓ DLPFC, temporal lobe activation
Page et al. (2009)	Go/No-go task	Adults	10 OCD patients (0 f) 11 Healthy controls (0 f)	Medication free Two patients met criteria for dysthymic disorder, three previously met criteria for depression and one previously met criteria for alcohol dependence	No Go > Go No Go > Go	↑ VMPFC posterior cingulate, premotor cortex, cerebellum ↓ OFC, DLPFC, ACC, putamen, caudate, hippocampus, thalamus
Rubia et al. (2010)	Stop-signal task	Adolescents	10 OCD patients (0 f) 20 Healthy controls (0 f)	Patients received treatment and were in partial remission No major psychiatric disorders, substance abuse, and learning disabilities	SS > Go FS > Go	↓ R. OFC (+correlation with improvement of symptoms) ↓ Left middle frontal gyrus
de Wit et al. (2012)	Stop-signal task	Adults	41 OCD patients (20 f) 17 Siblings (5 f) 37 Healthy controls (19 f)	Medication free Twenty-two patients met diagnostic criteria for another axis-I disorder. No psychosis, neurological illness, and severe medical conditions	SS > SG SS > SG	↑ Pre-SMA (also in unaffected siblings) ↓R. IFG and R. inferior parietal cortex
Kang et al. (2012)	Stop-signal task	Adults	18 OCD patients (6f) 18 Healthy controls (6f)	Medication free No major psychiatric disorders, psychosis, neurological illness, substance abuse, depression, and mental retardation	SS > Go SS > Go	↑ Bilateral superior parietal cortex, cerebellum, R. parahippocampal cortex ↓*R. putamen*, L. precentral gyrus, R. fusiform cortex, bilateral caudate and temporal lobe; R. middle occipital cortex, L. angular gyrus, L. cerebellum, and R. cingulate cortex

*ACC, anterior cingulate cortex; CBT, cognitive behavioral therapy; DLPFC, dorsolateral prefrontal cortex; f, female; FS, failed stop; IFG, inferior frontal gyrus; MTL, middle temporal lobe; OFC, orbitofrontal cortex; SS, successful stop; SSRI, selective re-uptake inhibitor; VMPFC, ventromedial prefrontal cortex*.

In a recent study, we examined functional connectivity during performance of the Stop-signal task in unmedicated adult OCD patients, their unaffected siblings, and healthy controls (van Velzen et al., under review). We performed psychophysiological interaction (PPI) analyses and DCM and found abnormal connectivity between the IFG and amygdala in patients and their siblings, suggesting that this pattern of connectivity is an endophenotype. Limbic activation may interfere with CSTC circuit activation in OCD. We did not find evidence for altered connectivity between the IFG, pre-SMA, and striatum during inhibition. These results warrant replication in other samples.

Two studies have investigated the effects of pharmacological treatment for OCD on response inhibition. Treatment with SSRI’s increased task-relevant brain activation during performance of an interference control task along with symptom improvement (Nakao et al., 2005b). However, due to the study design, it remains unknown if this change in activation occurs secondary to symptom improvement or due to the pharmacological treatment. A second study reported increased activation of multiple cortical and subcortical brain areas during a Go/No Go task in OCD patients treated with SSRIs compared to OCD patients who were not treated with SSRIs (Roth et al., 2007). However, this study was crossectional, included small patient groups and did not study the relationship with disease severity.

In summary, OCD patients show impairment in both interference control and motor response inhibition. Prefrontal and other brain areas within the CSTC circuits appear to be hyperactive during interference control, although results have been inconsistent. As task load increases during action restraint and action cancelation, CSTC areas generally become hypoactive compared with controls, although some compensation may occur. Decreased serotonin and increased dopamine transmission in CSTC circuits may underlie the response inhibition deficits. The presence of, and functional correlates of response inhibition deficits have also been investigated in disorders related to OCD, such as Tourette’s syndrome (TS), trichotillomania (TTM), and attention-deficit hyperactivity disorder (ADHD), enabling the disorder specificity of these cognitive dysfunctions and enabling comparison of these inhibition deficits across these disorders.

## Inhibition in Other Frontal–Striatal Disorders

### Tourette’s syndrome

Gilles de la Tourette’s syndrome, also known as Tourette’s syndrome, is a neurodevelopmental disorder characterized by motor tics and vocal tics (American Psychiatric Association, 2013). TS affects between 0.4 and 1% of the population (Swain et al., 2007; Robertson, 2008).

Like in OCD, dysfunction of the serotonergic and dopaminergic systems is implicated in the pathophysiology of TS [for a review, see Steeves and Fox (2008)]. Several clinical trials have shown that administration of dopamine antagonists, such as risperidone and haloperidol, are effective in suppressing tics in most patients (Bloch et al., 2011; Roessner et al., 2011). Neuroimaging studies have reported decreased availability of the D_2_ and D_3_-receptors in cortical (OFC, ACC, insula, temporal, and occipital cortex) and subcortical areas (thalamus and hippocampus) (Gilbert et al., 2006; Steeves et al., 2010) and increased striatal DaT availability (Malison et al., 1995; Muller-Vahl et al., 2000; Cheon et al., 2004; Serra-Mestres et al., 2004; Liu et al., 2010), although conflicting results have also been reported (Singer et al., 2002; Hwang et al., 2008). Neuroimaging of the serotonergic system in TS has shown increased binding of the serotonin 2A-receptor in many cortical (OFC, ACC, insula, temporal lobe, parietal lobe, and occipital lobe) and subcortical areas (thalamus, caudate, and hippocampus) (Haugbol et al., 2007) and increased SERT availability in the striatum and midbrain (Wong et al., 2008).

There is increasing evidence for frontal–striatal dysfunction in TS [for reviews, see Albin and Mink (2006) and Felling and Singer (2011)]. For instance, symptom severity correlated negatively with the degree of activation of CSTC circuits during tic suppression (Peterson et al., 1998) and prefrontal cortical thickness (Draganski et al., 2010) and volume of prefrontal CSTC areas was decreased in TS patients compared with healthy controls (Draganski et al., 2010).

More than 90% of all patients with TS also have co-morbid psychiatric disorders, most often OCD or ADHD (Robertson, 2011). It has been estimated that between 45 and 60% of TS patients suffer from OCD as well (Ghanizadeh and Mosallaei, 2009). As in OCD, many studies have investigated whether the involuntary motor symptoms in TS are related to motor response inhibition and interference control. Evidence for this, however, has been mixed; as some studies report impaired performance (Baron-Cohen et al., 1994; Crawford et al., 2005; Rankins et al., 2006; Channon et al., 2009; Eichele et al., 2010), especially with increasing task demands, while others do not (Ozonoff et al., 1994; Ozonoff and Jensen, 1999; Hershey et al., 2004; Verte et al., 2005; Watkins et al., 2005; Channon et al., 2006; Ray Li et al., 2006; Marsh et al., 2007; Raz et al., 2009; Sukhodolsky et al., 2010). These studies often included TS patients with co-morbid disorders and patients often used psychotropic medication. A meta-analysis of four studies using the Stop-signal task in TS found mild inhibitory deficits (Lipszyc and Schachar, 2010).

While evidence for behavioral impairment is not straightforward, inhibition-related brain activity seems to be altered in TS (see Table 3). With increasing age, patients with TS, compared with healthy controls, show increased recruitment of CSTC regions during interference control (Raz et al., 2009). Greater activation of CSTC areas, which was observed in TS patients during interference inhibition, might be considered a compensatory mechanism (Marsh et al., 2007). During motor response inhibition, patients with TS showed increased inhibition-related frontal brain activity in an event-related potential (ERP) study (Johannes et al., 2001). The authors noted that compensatory brain activation may explain why studies have not consistently observed response inhibition deficits in TS patients. No difference was found in brain activation between patients and controls during performance of a Go/No Go task, although the sample size was limited (Hershey et al., 2004). No study has yet investigated the direct effects of pharmacological treatment on behavioral or functional measures of response inhibition in TS, although, see Wylie et al. (2013).

**Table 3:** **Overview of fMRI studies that have used interference control tasks and response inhibition tasks in patients with Tourette’s syndrome**.

Study	Task	Age group	Participants	Medication and co-morbidities	Contrast	Findings in TS patients
Hershey et al. (2004)	Go/No-go task	Adults	8 TS patients (2 f) 10 Healthy controls	Medication-free (<24 h) Two patients with comorbid OCD, four patients with comorbid ADHD	Task > fixation	No differences in brain activation during task performance compared to controls
Raz et al. (2009)	Simon task	Children/adults	42 TS patients (16 f) 37 Healthy controls (17 f)	Medication use unreported One patient with comorbid OCD and one patient with comorbid OCD and ADHD	IC > C	↑ Activation of frontal–striatal regions with age in TS
Marsh et al. (2007)	Stroop task	Children/adults	66 TS patients (19 f) 70 Healthy controls (36 f)	Thirty-eight patients used psychoactive medication (haloperidol/risperidone/SSRIs) Twenty-five patients with comorbid ADHD; eight with comorbid ADHD, and five with comorbid OCD/ADHD	IC > C	↓ Deactivation of the mesial PFC and ventral ACC with age in TS patients Activation of the R. IFG, L. DLPFC, lenticular nucleus, and thalamus associated with better performance in controls and poorer performance in TS patients

*ACC, anterior cingulate cortex; C, congruent; DLPFC, dorsolateral prefrontal cortex; IC, incongruent; IFG, inferior frontal gyrus; f, female; L, left; PFC, prefrontal cortex; R, right; SSRI, selective serotonin re-uptake inhibitor*.

In summary, behavioral inhibitory deficits may be limited to a subgroup of Tourette’s patients, as compensatory brain activation during inhibition may conceal behavioral deficits in response inhibition in some patients. Increased dopamine transmission in CSTC circuits may underlie the deficits in response inhibition.

### Trichotillomania

Trichotillomania is an obsessive–compulsive related disorder (American Psychiatric Association, 2013). Patients with this disorder experience an urge to pull out their hair, which causes distress and functional impairment. Due to similarities between TTM and OCD, TTM was historically treated with SSRIs. Although initially considered effective in TTM (Stein et al., 1997), more recent studies report that SSRIs are ineffective in TTM (Streichenwein and Thornby, 1995; van Minnen et al., 2003) or only effective in a specific subgroup of TTM patients (Stanley et al., 1997a; Gadde et al., 2007). More recent clinical trials showed that treatment with atypical antipsychotics, such as olanzapine and aripiprazole, which exert their effects on among others, the serotonergic and dopaminergic system, are more promising (Van Ameringen et al., 2010; White and Koran, 2011).

It has been suggested that TTM symptoms originate from CSTC circuit dysfunction (Mataix-Cols and van den Heuvel, 2006). In support of this hypothesis, structural abnormalities in frontal areas and regions of the striatum have been observed in TTM (Chamberlain et al., 2008). As patients have difficulty suppressing a motor response, i.e., pulling out their hair, deficits in response inhibition may underlie the symptoms of this disorder (Chamberlain et al., 2006a).

Research on response inhibition in TTM is limited and conflicting. While performance of a related cognitive control task was unaltered, TTM patients showed deficits in interference control in the Stroop task (Stanley et al., 1997b; Bohne et al., 2005). Deficits in action cancelation have been reported, and the degree of impairment correlated with disease severity (Chamberlain et al., 2006a). Impairment in action restraint was limited to a distinct subgroup of patients with an early onset of the disorder (Bohne et al., 2008).

The neural or pharmacological substrates of response inhibition deficits in TTM have not yet been fully elucidated, as no inhibition-related neuroimaging studies have been performed in this patient group. Nor have there been any studies on the effects of pharmacological treatment on response inhibition. TTM patients do, however, exhibit structural abnormalities in CSTC circuit regions associated with inhibition, for instance in the striatum, IFG, SMA, and prefrontal areas (Grachev, 1997; O’Sullivan et al., 1997; Chamberlain et al., 2008), which may underlie response inhibition impairment in TTM.

### Attention-deficit hyperactivity disorder

Attention-deficit hyperactivity disorder is a neuropsychiatric disorder characterized by hyperactivity, inattentiveness, and impulsiveness (American Psychiatric Association, 2013). It is a common disorder, as it is thought to affect almost 10% of school-aged children (Froehlich et al., 2007). The neuropharmacology of ADHD is complex and still not well-understood. Current evidence suggest that ADHD is characterized by deficits in the noradrenalin and dopamine systems [for a review, see McAlonan et al. (2009)], although some studies show additional involvement of the serotonergic (Oades et al., 2002) system. Pharmacotherapeutic treatment of ADHD with methylphenidate, amphetamines, or atomoxetine is effective in treating symptoms, presumably through increasing extracellular levels of dopamine and noradrenalin [see Prince (2008) for a review].

Patients with ADHD show behavioral impairments on a number of interference control tasks, including the Simon task and the Flanker task (Rubia et al., 2011a; Sebastian et al., 2012). Activation of the ACC, IFG, thalamus, SMA, striatum, and inferior parietal cortex is decreased in ADHD patients during interference control (see Table 4). A recent meta-analysis revealed decreased activation of CSTC areas, including the right IFG, insular cortex, right caudate nucleus, left inferior parietal cortex, and left ACC in ADHD patients during performance of the Stroop and the Simon task (Hart et al., 2012).

**Table 4:** **Overview of fMRI studies that have used interference control tasks in patients with attention-deficit hyperactivity disorder**.

Study	Task	Age group	Participants	Medication and co-morbidities	Contrast	Findings in ADHD patients
Vaidya et al. (2005)	Modified Flanker task	Children	10 ADHD patients (3 f) 10 Healthy controls (3 f)	Medication-naïve or medication free (36 h) Symptoms of ODD present in seven patients; symptom of CD reported in two children	IC > N	↓ L. IFG
Vasic et al. (2012)	Modified Flanker task	Adults	14 ADHD patients (0 f) 12 Healthy controls (0 f)	Medication free (4 days) No comorbid psychiatric disorders, substance abuse, neurological disorders, learning disabilities	Error > correct	↓ L. IFG during error processing
Cubillo et al. (2011)	Simon task	Adults	11 ADHD patients (0 f) 15 Healthy controls (0 f)	Medication-naive Three patients had ADHD symptoms, but did not meet all criteria for ADHD. Comorbid disorders: one patient with anxiety, three with mood disorders, one with CD, and two with substance abuse	IC > C	↓ L. IFG/OFC, L. medial frontal cortex, L. ACC, L. caudate, L. premotor cortex
Rubia et al. (2011b)	Simon task	Children	12 ADHD patients (0 f) 13 Healthy controls (0 f)	Medication-naïve One patient met criteria for ODD/CD	IC > oddball	↓R. IFG, R. IPC, L. VMPFC, basal ganglia, thalamus, R. SMA/ACC/posterior cingulate, L. superior/middle temporal/occipital cortex
Rubia et al. (2011a)	Simon task	Children	18 ADHD patients (0 f) 20 Healthy controls (0 f)	Medication-naïve One patient met criteria for CD	IC > oddball	↓ R. SMA/ACC/superior parietal lobe, R. IPC
Sebastian et al. (2012)	Simon task	Adults	20 ADHD patients (9 f) 24 Healthy controls (13 f)	Unmedicated or medication-free (2 months) Eight patients with current comorbid disorders (dysthymia, anxiety disorders, substance abuse, and personality disorders)	IC > C	↓ R. precentral gyrus, L. paracentral lobe, L. middle cingulate cortex, bilateral superior temporal gyrus, L. middle temporal gyrus, R. temporal pole, *R. insula, R. pallidum*
Bush et al. (1999)	Stroop task	Adults	8 ADHD patients (3 f) 8 Healthy controls (3 f)	Medication-free (>48 h) No comorbid psychiatric disorders, neurological disorders, learning disability, and medical illness	IC > N	↓ACC (cognitive division)
Smith et al. (2006)	Stroop task	Children/adolescents	17 ADHD patients (0 f) 18 Healthy controls (0 f)	Medication-naïve Five patients with comorbid conduct disorder	IC > oddball	No significant differences
Banich et al. (2009)	Stroop task	Adults	23 ADHD patients (9 f) 23 Healthy controls (10 f)	Medication-free (24 h) No comorbid psychiatric disorders, learning disability, history of seizures, or head injury	IC > N IC > C	↓ L. supramarginal gyrus ↑ *R. cuneus*, R. middle frontal gyrus
Peterson et al. (2009)	Stroop task	Adolescents	16 ADHD patients (3 f) 20 Healthy controls (8 f)	Medication-free Five patients had comorbid disorders (ODD, depression, anxiety disorders, and phobias)	IC > C	↓ L. ACC, *L. insula*, R. precuneus, thalamus, and caudate ↑ R. hippocampus, R. superior frontal gyrus, and L. ACC
Burgess et al. (2010)	Stroop task	Adults	20 ADHD patients (8 f) 23 Healthy controls (10 f)	Medication free (24 h) No comorbid psychiatric or learning disorder	IC > N	↑ R. superior frontal gyrus

*ACC, anterior cingulate cortex; C, congruent trials; CD, conduct disorder; IC, incongruent trials; IFG, inferior frontal gyrus; IPC, inferior parietal cortex; f, female; L, left; N, neutral trials; ODD, oppositional defiance disorder; OFC, orbitofrontal cortex; R, right; SMA, supplementary motor area; VMPFC, ventromedial prefrontal cortex*.

Motor response inhibition is also affected in ADHD. A meta-analysis of 24 Stop-signal paradigm studies showed increased SSRT and mean GO reaction times in ADHD patients (Alderson et al., 2007). Structural abnormalities have been observed in CSTC circuit areas, including the IFG, caudate, and globus pallidus (Durston, 2003; Sowell et al., 2003; Batty et al., 2010; Depue et al., 2010; Frodl and Skokauskas, 2012), leading some to argue that altered brain structure of these areas may underlie the impairments in response inhibition (Chambers et al., 2009). Gray matter volume of the right IFG, ACC, caudate nucleus, medial temporal lobe, and globus pallidus correlated negatively with task performance in patients (Casey et al., 1997; McAlonan et al., 2009). Functional neuroimaging studies show reduced inhibition-related activation of the caudate nucleus, IFG, and SMA, and increased activation of areas in the temporal and parietal lobe in children and adults with ADHD (see Table 5; Schulz et al., 2004; Tamm et al., 2004; Booth et al., 2005; Rubia et al., 2005b; Suskauer et al., 2008; Dibbets et al., 2009; Dillo et al., 2010; Kooistra et al., 2010; Mulligan et al., 2011; Spinelli et al., 2011). A recent meta-analysis of 21 response inhibition studies revealed hypoactivation of the right ACC, right IFG, right insular cortex, right thalamus, left caudate nucleus, and right fusiform gyrus (Hart et al., 2012). In addition to decreased frontal–striatal connectivity, altered frontal–parietal connectivity may also play a role in the response inhibition impairment of ADHD (Cubillo et al., 2010).

**Table 5:** **Overview of fMRI studies that have used response inhibition tasks in patients with attention-deficit hyperactivity disorder**.

Study	Task	Age group	Participants	Medication and co-morbidities	Contrast	Findings in ADHD patients
Rubia et al. (1999)	Stop-signal task	Adolescents	7 ADHD patients (0 f) 9 Healthy controls (0 f)	Medication-naïve or medication-free (1 week) No comorbid psychiatric disorder (except conduct disorder) or neurological disease	Stop > Go	↓ R. IFG, R. MPFC, L. caudate
Rubia et al. (2005b)	Stop-signal task	Adolescents	16 ADHD patients (0 f) 21 Healthy controls (0 f)	Medication-naïve Five patients with conduct disorder. No neurological disease, substance abuse or previous treatment with stimulants	SS > FS FS > Go	↓ R. frontotemporal pole, R. OFC, R. superior temporal lobe ↓ R. Posterior cingulate/precuneus
Pliszka et al. (2006)	Stop-signal task	Children/adolescents	9 Treated ADHD patients (1f) 8 Medication-free ADHD patients (3 f) 15 Healthy controls (6 f)	Medication-naïve or medication free No psychiatric disorder (except ODD), substance abuse, alcohol abuse	Stop > Go	↑ R. DLPFC
Cubillo et al. (2010)	Stop-signal task	Adults	11 Adults with persistent ADHD (0 f) 14 Healthy controls (0 f)	Medication-naïve Seven subjects with axis-I disorders (anxiety, depression, conduct disorder, substance related disorders). No neurological abnormalities, treatment with stimulants	SS > Go FS > Go	↓ L. IFG/insula, R. IFG/insula, striatum, thalamus, R. premotor cortex, bilateral SMA/ACC ↓R. IFG/insula, thalamus, striatum
Passarotti et al. (2010)	Stop-signal task	Children/adolescents	11ADHD patients (5 f) 15 Healthy controls (8 f)	Medication-naïve or medication-free (1 week) No comorbid psychiatric conditions, neurological disorders, learning disabilities, history of substance abuse	Stop > Go	↑ L. caudate, R. caudate tail, L. cerebellum ↓ R. middle, superior and inferior frontal gyrus, L. superior and inferior frontal gyrus; L. superior temporal gyrus
Rubia et al. (2011c)	Stop-signal task	Children	12 ADHD patients (0 f) 13 Healthy controls (0 f)	Medication-naive One patient with comorbid ODD/CD. No psychiatric disorders, learning disabilities, neurological disorders, epilepsy, substance abuse, treatment with stimulants	FS > Go SS > Go	↓ L. IFG, Pre-SMA, R. premotor cortex, bilateral thalamus, R. IPC, L. posterior cingulate, L. precuneus, cerebellum ↓ Bilateral IFG, bilateral pre-SMA, thalamus, bilateral ACC, R. IPC/precuneus/posterior cingulate, cerebellum
Sebastian et al. (2012)	Stop-signal task	Adults	20 ADHD patients (9 f) 24 Healthy controls (13 f)	Unmedicated or medication-free (2 months) Eight patients with current comorbid disorders (dysthymia, anxiety disorders, substance abuse, personality disorders)	Stop > Go SS > FS	↓ R. Pallidum ↓ L.IFG, bilateral putamen, R. caudate, *L. insula, L. pallidum*
Durston et al. (2003)	Go/No-go task	Children	7 ADHD patients (1 f) 7 Healthy controls (1 f)	Medication-free (1 day) Comorbid disorders not reported	No Go > Go	↓ L. caudate ↑ R. Middle and superior frontal gyrus, L. IPC, bilateral posterior cingulate/precuneus, R. superior temporal gyrus
Tamm et al. (2004)	Go/No-go task	Adolescents	10 ADHD patients (0 f) 12 Healthy controls (0 f)	Medication-naïve and medication free (18 h) Controls had no family history of psychiatric disorders, no neurological or developmental disorders	No Go > Go	↓ R. ACC/SMA, R. superior and middle frontal gyrus ↑ L. superior/middle/inferior temporal gyrus
Schulz et al. (2004)	Go/No-go task	Adolescents	10 Individuals with childhood ADHD diagnosis (0 f) 9 Healthy controls (0 f)	Medication-free (6 months) One patient with conduct disorder	No Go > Go	↑ Bilateral IFG, bilateral middle frontal gyrus, L. ACC, bilateral IPC, right precuneus ↓ R. precentral gyrus, R. inferior temporal gyrus, L. hippocampus, bilateral cerebellum
Booth et al. (2005)	Go/No-go task	Children	12 ADHD patients (4 f) 12 Healthy controls (5 f)	Medication-free (2 days) No comorbid psychiatric disorders, neurological disorders, substance abuse, visual or hearing impairment	No Go > Go	↓ R. IFG, R. superior frontal gyrus, medial frontal gyrus, bilateral caudate, *R. amygdala*, thalamus, fusiform gyrus, *L. cuneus, L. globus* pallidum
Smith et al. (2006)	Go/No-go task	Children/adolescents	17 ADHD patients (0 f) 18 Healthy controls (0 f)	Medication-naïve	No Go > oddball go Five patients with comorbid conduct disorder	↓ L. rostral mesial frontal cortex
Suskauer et al. (2008)	Go/No-go task	Children/adolescents	25 ADHD patients (10 f) 25 Healthy controls (10 f)	Medication-free (2 days) Eleven patients also met criteria for ODD, five patients met criteria for specific phobia, two controls met criteria for specific phobia	No Go	↑ R. precentral gyrus ↓R. ACC, L. precentral gyrus, *L. putamen*, R. temporal–parietal junction, R. fusiform gyrus, L. precuneus, L. posterior cingulate, L. cerebellum
Dibbets et al. (2009)	Go/No-go task	Adults	16 ADHD patients (0 f) 13 Healthy controls (0 f)	Medication-free (24 h) Two patients with depressive symptoms, one reported OCD symptoms, two reported learning disabilities and one reported substance abuse	Go No Go	↑ R. middle frontal gyrus, L. IFG ↑ L. IFG, *R. putamen*
Dillo et al. (2010)	Go/No-go task	Adults	15 ADHD patients (4 f) 15 Healthy controls (4 f)	Medication-free (3 weeks) No comorbid psychiatric diagnosis, substance abuse, neurological disorders	No Go > Go	↑ Bilateral inferior/superior parietal lobe, left inferior/middle occipital gyrus
Kooistra et al. (2010)	Go/No-go task	Adults	10 ADHD patients (0 f) 10 Healthy controls (0 f)	Medication-naive Two patients in partial remission, no comorbid psychiatric disorders, neurological disorders, cognitive impairment, motor disabilities	No Go > Go	↑ R. supramarginal gyrus, R. ACC
Mulligan et al. (2011)	Go/No-go task	Adults	12 ADHD patients (0 f) 12 Healthy controls (0 f)	Medication free (>2 days) No comorbid axis-I diagnosis, history of learning disability, history of neurological disorders, alcohol or substance dependence, use of stimulants	No Go	↓ R. Pre-SMA, bilateral IPC, L. precentral gyrus, R. frontal eye fields, L. precuneus
Spinelli et al. (2011)	Go/No-go task	Children	13 ADHD patients (4 f) 17 Healthy controls (9 f)	Medication free (2 days) Three patients had comorbid ODD, one a specific phobia	Post error > Post correct	↑ R. superior frontal gyrus, L. medial frontal gyrus, R. cingulate gyrus, R. postcentral gyrus, R. inferior/middle temporal gyrus
Sebastian et al. (2012)	Go/No-go task	Adults	20 ADHD patients (9 f) 24 Healthy controls (13 f)	Unmedicated or medication-free (2 months) Eight patients with dysthymia, anxiety disorders, substance abuse	Stop > Go	↓ R. caudate

*ACC, anterior cingulate cortex; DLPFC, dorsolateral prefrontal cortex; FS, failed stop-trials; IFG, inferior frontal gyrus; IPC, inferior parietal cortex; f, female; L, left; MPFC, medial prefrontal cortex; ODD, oppositional defiance disorder; OFC, orbitofrontal cortex; Pre-SMA, pre-supplementary motor area; R, right; SMA, supplementary motor area; SS, successful stop-trials*.

Pharmacological studies show that administration of methylphenidate and atomoxetine improve action cancelation (Aron et al., 2003a; Chamberlain et al., 2007a; DeVito et al., 2009; Coghill et al., 2013) and action withholding (Vaidya et al., 1998) in ADHD patients, thereby suggesting that deficits in dopamine and noradrenalin underlie motor response inhibition deficits. Furthermore, use of methylphenidate increased prefrontal and striatal activation during performance of a Go/No Go task in ADHD patients (Vaidya et al., 1998). Methylphenidate also normalizes activation deficits in prefrontal, parietal, temporal, and cerebellar regions during performance of the Stop-signal task (Rubia et al., 2011b). When effects of atomoxetine and methylphenidate were directly compared, both medications normalized left prefrontal underactivation during performance of the stop-signal task, while normalization of the right prefrontal activation was specific to use of methylphenidate (Cubillo et al., 2014).

Several candidate gene studies report on an association between genotype and response inhibition deficits in ADHD. In patients with ADHD, a polymorphism of the DRD4 gene, which is associated with decreased functional activity of the dopamine D_4_-receptor (Asghari et al., 1995), was related to altered performance on tasks with an inhibitory component (Langley et al., 2004; Bellgrove et al., 2005), impaired performance on the Stroop task (Loo et al., 2008), and reduced prefrontal cortical thickness (Shaw et al., 2007).

Attention-deficit hyperactivity disorder patients homozygous for a polymorphism of the DaT gene associated with increased transporter expression (Brookes et al., 2007), showed increased frontal and parietal brain activation during a modulated Go/no-go task (Braet et al., 2011), and showed increased activation in the striatum, premotor, and parietal cortices during inhibition in the Go/No-go task (Bedard et al., 2010). In contrast, a second study found increased striatal activity during inhibition in polymorphisms that result in decreased function (Durston et al., 2008).

In individuals with a specific polymorphism of the MAO-A gene, associated with lower levels of MAO-A, ADHD symptoms were related to decreased IFG activation during the Stop-signal task (Nymberg et al., 2013). MAO-A genotype of ADHD patients was not related to interference inhibition (Liu et al., 2011).

In summary, behavioral deficits in interference control and motor response inhibition are prominent in ADHD and associated with decreased volume and hypoactivation of CSTC areas. Results of gene-association studies suggest that reduced inhibitory performance may be related to decreased dopamine transmission in CSTC circuits.

## Comparison between and Integration Across Disorders

**Figure 2:**
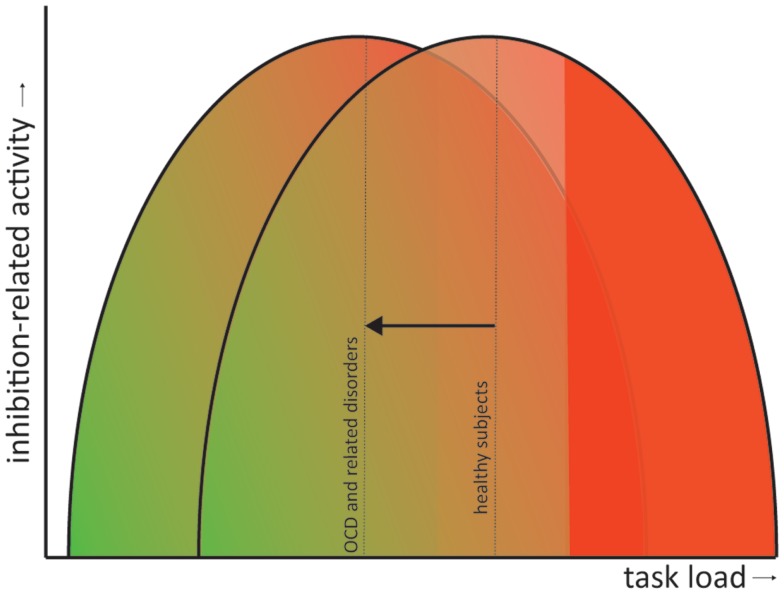
**Shift in the inverted *U*-shaped relation between task load and inhibition-related activity**. Inhibition-related neural circuit activity gradually increases with task load (green to red gradient). However, when task demands become too high the compensatory activity starts to fail and behavioral performance becomes impaired (solid red). In obsessive– compulsive and related disorders performance impairments and failure of compensatory neural activation occur at a lower task load than in healthy controls (visualized as a shift of the inverted *U*-shaped curve to the left).

All discussed disorders exhibit symptoms that signify a failure to inhibit certain impulses or responses. Response inhibition tasks therefore provide a very good operationalization to study the neural correlates of some dysfunctions contributing to the symptomatology of these disorders. From the above reviewed literature we can conclude that, overall, patients with obsessive–compulsive or related disorders exhibit deficits in response inhibition concomitant with alterations in the task-related brain activity. Whether these brain areas are *hypo*- or *hyper*activated compared with matched healthy controls depends largely on the complexity of the task. In general, we can state that patients compensate behavior by recruiting additional inhibition-related brain areas, explaining why behavioral performance is often normal, but only during less complex tasks (e.g., the Flanker task and the Simon task). With increasing task demand (e.g., the Go/no-go and the Stop-signal task), these compensational mechanisms fail and patients start to show behavioral impairments and decreased inhibition-related neural circuit activity. This phenomenon has also been observed in healthy subjects (Sebastian et al., 2013a), although they can “endure” tasks with higher demands before overstressing the inhibition network and concomitant decrements in performance. In other words, patients exhibit performance impairments and failure of compensatory activation at a lower task load than healthy controls. This has also been observed in OCD patients and their siblings and adult patients with ADHD while performing a working memory task such as the N-Back (de Vries et al., 2013; Ko et al., 2013). Figure 2 illustrates this as a shift to the left of an inverse *U*-shape relation between task load and inhibition-related activity. This shift in compensatory abilities does not have to be specific for the discussed disorders but may also apply to others, such Parkinson’s disease (Vriend et al., under review) or even natural aging (Sebastian et al., 2013a).

The actual neurobiological mechanism for this shift is, unfortunately, less apparent and may involve (interactions between) electrophysiological anomalies, neurotransmitter dysfunction, genetic variance, etc. A prime candidate for the cause of the shift might by dysfunction of dopamine signaling. Dopamine is the major neuromodulator in the CSTC circuits and can either facilitate or inhibit their activation depending on the activation of their different receptor subtypes and dopamine concentrations (Alexander et al., 1986; Vriend et al., 2014).

**Figure 3:**
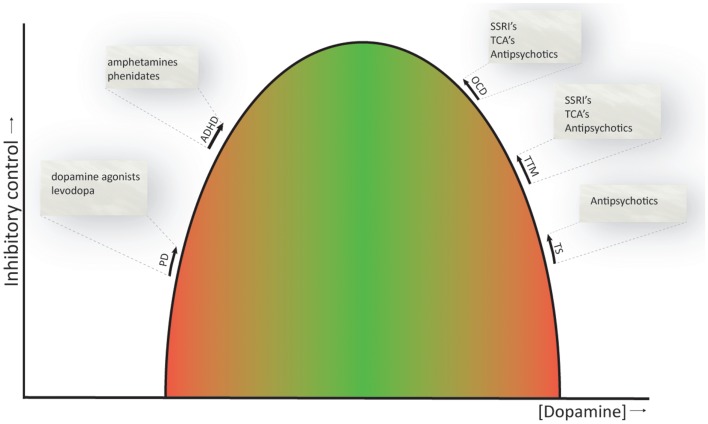
**Inverted *U*-shaped relation between dopamine levels and inhibitory control**. The ability to control behaviors, impulses, and urges is influenced by dopamine, and both reduced and increased dopamine levels (green to red gradient) have a detrimental effect on inhibitory control. Current evidence suggests that ADHD is a *hypo*dopaminergic disorder, while OCD, TTM, and TS are considered *hyper*dopaminergic disorders. Inhibitory deficits are also evident in patients with Parkinson’s disease, a prototypical *hypo*dopaminergic disease. Pharmacotherapeutics used to treat the symptoms of these disorders are listed and are thought to normalize dopamine levels and thereby ameliorate response inhibition (indicated by the arrows). PD, Parkinson’s disease; ADHD, attention-deficit hyperactivity disorder; OCD, obsessive–compulsive disorder; TTM, trichotillomania; TS, Tourette’s syndrome. NB. Since comparison studies across OCD, TTM, and TS are in short supply, the spacing between these disorders on the *U*-shaped curve is arbitrary and does not necessarily represent actual differences in dopamine levels between these disorders.

As reviewed above, the CSTC circuits seem to be important for response inhibition (Aron, 2011) and are also involved in the pathophysiology underlying the dysfunctions related to the symptomatology of the obsessive–compulsive and related disorders. Whether or not dopamine is primarily involved in the pathophysiology of these disorders is still under debate, with some of the above reviewed studies showing clear associations, while others do not. Nevertheless, current evidence suggests that ADHD can be seen as a *hypo*dopaminergic disorder, whereas OCD, TTM, and TS can be regarded as *hyper*dopaminergic disorders (Buse et al., 2013). This is also consistent with the currently available pharmacological treatments, whose neurobiological mechanism is thought to rely on restoring dopamine to physiological levels (Abi-Dargham and Laruelle, 2005; Gerlach et al., 2013). Even SSRI’s and tricyclic antidepressants, the first line pharmacological treatment of OCD, may normalize dopamine levels by upregulation of serotonin signaling, that has an inhibitory effect on dopamine (Boureau and Dayan, 2010). Figure 3 provides a schematic representation of the proposed relation between dopamine levels and inhibitory control. This relation is similar to the inverse *U*-shaped relation proposed for dopamine levels and working memory function (Cools and D’Esposito, 2011).

The proposed relation obviously does not provide the full story and is merely intended as a framework to understand some of the findings discussed in this review. Dopamine has differential effects in the prefrontal cortex and striatum, different firing modes (i.e., tonic and phasic) and highly complex interactions with other neurotransmitter systems, including the serotonin, noradrenalin, and glutamate system, and even hormones, such as estrogens (Boureau and Dayan, 2010; Cools and D’Esposito, 2011; de Bartolomeis et al., 2013), which prohibits a clear understanding of the influence of these neurotransmitters on brain activity and behavior.

In short, the functional and behavioral deficits in response inhibition in obsessive–compulsive and related disorders can be conceptualized as a shift in the relation between task demands and inhibition-related neural circuit activity. What causes this shift and what could thereby underlie the symptoms of these disorders is currently unknown, although we postulate that dopamine plays a critical role.

## Conclusion and Future Directions

The aim of this review was to provide an overview of the studies that examined the neural, pharmacological, and genetic substrates of inhibitory impairment of disorders within the impulsive–compulsive spectrum, with a focus on OCD, ADHD, TS, and TTM. We have shown that functionally and behaviorally impaired response inhibition is a shared characteristic among these disorders and may underlie at least some of the dysfunctions related to the symptomatology of the disorders. Neuroimaging studies suggest that inhibition-related brain areas are mostly hypoactivated in ADHD and OCD (although dependent on the task load), while studies in TS have provided mixed results. To our knowledge, no study has yet been published on the neural correlates of response inhibition in TTM. Dopamine and serotonin signaling seems to be important for response inhibition and dysfunction of these neurotransmitters has been frequently observed in the obsessive–compulsive and related disorders. Nevertheless, almost all imaging studies on neurotransmitters have been performed in patients that received (chronic) pharmacotherapy, which may have influenced the scan directly, due to competition of the drug with a radioligand for a specific binding site, or indirectly because the brain adapts to the pharmacological effects (Wang et al., 2013). For a better understanding of the pathophysiology of the disease itself and the identification of novel treatment targets, more studies are needed in medication-naïve patients. Prospective follow-up of these patients after commencing treatment can subsequently provide insights into the effect of treatment on response inhibition impairments and its relation to disorder-specific symptoms. Lastly, there is a relative lack of studies that compare the pathophysiology of inhibitory deficits across related mental disorders. Such studies allow the identification of common as well as specific disease biomarkers of impulsivity and compulsivity symptoms.

## Author Contributions

All authors were involved in design of this review; Laura S. van Velzen and Chris Vriend drafted the first version of the manuscript; all authors contributed to the interpretation of the literature, revised earlier drafts of this article and have approved the final version of the manuscript and its submission.

## Conflict of Interest Statement

The authors declare that the research was conducted in the absence of any commercial or financial relationships that could be construed as a potential conflict of interest.
